# A unifying modeling of plant shoot gravitropism with an explicit account of the effects of growth

**DOI:** 10.3389/fpls.2014.00136

**Published:** 2014-04-14

**Authors:** Renaud Bastien, Stéphane Douady, Bruno Moulia

**Affiliations:** ^1^Institut Jean-Pierre Bourgin, UMR1318 INRA-AgroParisTechVersailles, France; ^2^UMR 547 PIAF, INRAClermont-Ferrand Cedex 01, France; ^3^UMR 547 PIAF, BP 10448, Clermont Université, Université Blaise PascalClermont-Ferrand, France; ^4^Matière et Systèmes Complexes, Université Paris-DiderotParis Cedex 13, France; ^5^Department of Physics, School of Engineering and Applied Sciences, Harvard UniversityCambridge, MA, USA

**Keywords:** gravitropism, growth, proprioception, morphogenesis, control

## Abstract

Gravitropism, the slow reorientation of plant growth in response to gravity, is a major determinant of the form and posture of land plants. Recently a universal model of shoot gravitropism, the *AC* model, was presented, in which the dynamics of the tropic movement is only determined by the conflicting controls of (1) graviception that tends to curve the plants toward the vertical, and (2) proprioception that tends to keep the stem straight. This model was found to be valid for many species and over two orders of magnitude of organ size. However, the motor of the movement, the elongation, was purposely neglected in the *AC* model. If growth effects are to be taken into account, it is necessary to consider the material derivative, i.e., the rate of change of curvature bound to expanding and convected organ elements. Here we show that it is possible to rewrite the material equation of curvature in a compact simplified form that directly expresses the curvature variation as a function of the median elongation and of the distribution of the differential growth. By using this extended model, called the *ACĖ* model, growth is found to have two main destabilizing effects on the tropic movement: (1) passive orientation drift, which occurs when a curved element elongates without differential growth, and (2) fixed curvature, when an element leaves the elongation zone and is no longer able to actively change its curvature. By comparing the *AC* and *ACĖ* models to experiments, these two effects are found to be negligible. Our results show that the simplified *AC* mode can be used to analyze gravitropism and posture control in actively elongating plant organs without significant information loss.

## Introduction

**Figure 1:**
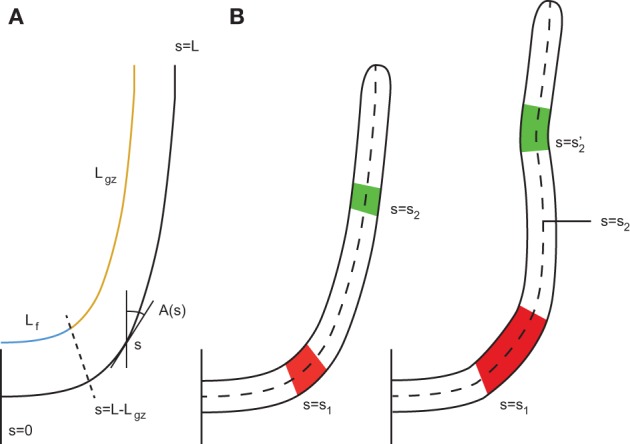
**Geometric description of a rod-like plant organ. (A)** The arc length *s* is defined along the median line of the organ with *s* = 0 referring to the base and *s* = *L* the apex. The total length of the organ *L* can be broken down into the length of the growth zone *L*_*gz*_, which is moving with the apex, and *L*_*f*_, the length of the zone which is not growing. This fixed portion elongates with time as elements leave the active growth zone. *A*(*s*) is the local orientation with respect to the vertical. **(B)** To simplify this, only two parts of the organ are considered to be elongating, the red part, at a position defined at its base *s* = *s*_1_, and the green part, at a position defined at its base *s* = *s*_2_. During elongation, the curvatures of these two parts are modified. Due to elongation, their distance relative to each other is modified. The position *s* = *s*_2_ no longer refers to the green element. To account for the variation in the material element, the derivative must follow the material element.

Plant gravitropism is the capacity of plants to reorient themselves according to the gravitational field. The control over these movements involves several types of perception. In shoots, gravity is perceived by specialized cells all along the growth zone (Moulia and Fournier, 2009; Morita, 2010). This perception is then converted into a change in curvature of the organ powered by the differential growth mediated by enhanced lateral transport of the plant hormone auxin (Silk, 1984; Moulia and Fournier, 2009). Graviception has been an intensive field of study for more than a century (Sachs, 1888; Moulia and Fournier, 2009; Morita, 2010). However, it is only recently that kinematic studies have shown the universal importance of the straightening movement of organs where the sensing of curvature is local, e.g., aerial shoots (Bastien et al., 2013). This form of growth has been described in a simple model, called the *AC* model. The *AC* model revealed that the control of the gravitropic movement involves not only graviception, but also proprioception of the organ curvature. For small curvatures, this model is written

(1) $$\frac{\partial C(s,t)}{\partial t}=-\beta \sin A(s,t)-\gamma C(s,t)$$

where *t* is time, *s* is the curvilinear abscissa along the organ defined from the base *s* = 0 to the apex *s* = *L*(*t*), *L*(*t*) is the total length of the organ, *A*(*s, t*) is the angle of the material element at position *s* and time *t* with respect to the vertical, and $C(s,t)\;=\;\frac{\partial A(s,t)}{\partial s}$ is the curvature of this material element (Figure 1). The proprioceptive term, −γ*C*(*s, t*), tends to keep the plants straight. The graviceptive term −β sin *A*(*s, t*) follows the sine law of gravitropism (Moulia and Fournier, 2009; Bastien et al., 2013) and tends to align the plant with the direction of the gravity field. When organ alignment approaches the vertical, near *A*(*s, t*) = 0, the graviceptive term can be linearized, β sin *A*(*s, t*) ~ β*A*(*s, t*), yielding the most simple form of the *AC* model

(2) $$\frac{\partial C(s,t)}{\partial t}=-\beta A(s,t)-\gamma C(s,t)$$

The graviproprioceptive *AC* model (Equation 2) is both minimal and efficient in describing the observed universal pattern of curvature (Bastien et al., 2013). Equation (2) has been proposed as a way to phenotype gravitropism in shoot organs of plants, through the use of the dimensionless number *B* = β*L*_*gz*_/γ, where *L*_*gz*_ is the length of the zone that senses gravity and curvature and is able to undergo active bending. The number *B* quantifies the ratio between graviceptive and proprioceptive terms and entirely controls both the transient dynamics and the final steady-state shape of the gravitropic organ. When *B* was defined initially, the subscript *gz* was coined for “growth zone,” as the gravitropic response usually occurs within the growth zone (Morita, 2010). However, it is important to note that in the *AC* model the motor of the movement, i.e., the growth, was not explicitly specified. The movement was assumed to take place without any mean elongation so that the total length of the organ, *L*, and of the growth zone, *L*_*gz*_, remained constant.

To move on from these assumptions to a more realistic description, it is important to extend the *AC* model by introducing the motor of change of curvature, i.e., the differential growth across the stem, and the mean elongation (as plant cells in the primary growth zone do not shrink actively). To take into account the effects of growth, it is necessary to consider the change in curvature following the material points and not just the external shape even if this shape is stationary (Silk and Erickson, 1978; Chavarria-Krauser, 2006; Moulia and Fournier, 2009; Merret et al., 2010) (Figure 1). In addition, through the elongation of the cells, the distance between material elements is continuously modified, and thus the orientation of the elements may change accordingly (Silk and Erickson, 1978).

Here we investigate the consequences of including the mean elongation and differential elongation in modeling the control of the gravitropic movement of shoots. First, we extend the *AC* model to incorporate the effects of growth. As plants regulate curvature variation through differential growth, it would be useful to be able to express the visible output, the variation in curvature, as a function of differential growth. In order to properly identify the effects of growth a simplified compact expression of this relationship is derived here. Second, the striking features of the *AC* model including elongation are explored. Two cases of elongation are studied, when the whole organ is elongating and when only the subapical part is growing. The destabilizing effects of these two types of growth are identified. The conditions for which the basic graviproprioceptive *AC* model (without growth) (Bastien et al., 2013) can account for the behavior of growing organs are discussed. Comparison with experimental data will give an estimation of the validity of these approximations.

## Results

### Kinematic relationship between curvature variation and growth

**Figure 2:**
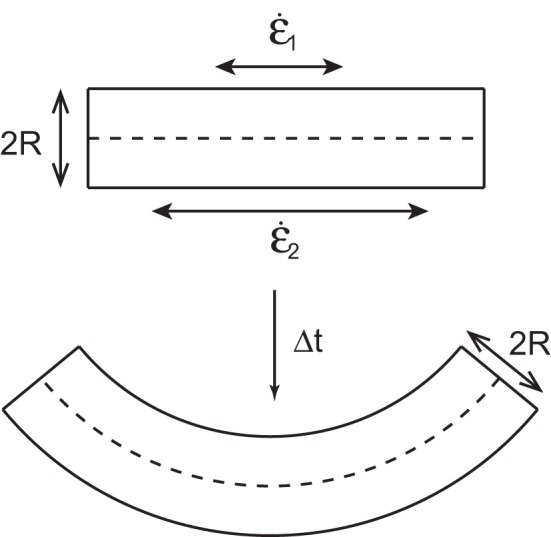
**A small element of an organ is considered as a cylinder of radius R with the upper side of length δ*s*_1_ and the lower side of length δ*s*_2_**. The sides are growing with a relative elongation rates of $\dot{\epsilon }$_1_ and $\dot{\epsilon }$_2_, respectively. After time *t* the curvature of the organ has changed.

When modeling an organ a given segment of the organ is approximated by a cylinder with a radius *R* (Figure 2). This is a sufficient approximation for the description of subapical plant movement like elongation and bending but not for apical growth. Gravitropic movement has been shown experimentally to lay within a single vertical plane defined by the initial orientation of the organ and the vertical. The curvature C of the organ thus lays within this plane (Darwin and Darwin, 1897; Correll and Kiss, 2008; Moulia and Fournier, 2009). Within this plane both sides of the organ are growing with relative elongation growth rates, designated, respectively as $\dot{\epsilon }$_1_ and $\dot{\epsilon }$_2_. From the simple geometrical model in Figure 2, the temporal evolution of the curvature of a material element with differential growth rate is given by

(3) $$\frac{DC(s,t)}{Dt}=\frac{1}{2R}(1-C{\left(s,t\right)}^{2}R^{2})({\dot{\epsilon }}_{2}-{\dot{\epsilon }}_{1})$$

assuming there is no shear growth in plants [a plane cross section remains plane despite bending, fulfilling the Navier Bernouilli specification of the rod mechanics theory (Doghri, 2000), (see Supplementary Material for more details)]. As an organ expands, segments are moved along the organ relative to each other. So the material derivative comoving with each element of the organ has to be taken into account (see Silk, 1984; Moulia and Fournier, 2009 for more details). With respect to the curvilinear abscissa along the median line of the organ from the base to the apex (Figure 1), this material derivative is defined by

(4) $$\frac{DC(s,t)}{Dt}=\frac{\partial C(s,t)}{\partial t}+v(s,t)\frac{\partial C(s,t)}{\partial s}$$

where *v*(*s, t*) is the velocity of the growth-induced displacement of each element along the organ. This is strictly equivalent to the expression given by Silk (1984), the first formula to express the relationship between curvature variation and differential growth (Supplementary Material). Another equation has been shown to be equivalent to the Silk equation up to the second order in *C*(*s, t*)*R* (Chavarria-Krauser, 2006). These three equations are thus equivalent, at least, for small curvatures. But the formulation of Equation (3) is advantageous here as it expresses the curvature variation directly as the difference in elongation on each side of the organ. If $\dot{\epsilon }$_1_ + $\dot{\epsilon }$_2_ is not zero, i.e., if the organ undergoes net elongation, then we can separate the effects of the mean elongation rate of the segment, and of the relative distribution of differential growth across the organ as

(5) $$\frac{DC(s,t)}{Dt}=\frac{1}{R}\left(1-C{\left(s,t\right)}^{2}R^{2}\right)\dot{E}(s,t)\Delta (s,t)$$

where *Ė*(*s, t*) is the relative elemental elongation rate on the median line defined as the mean of the elongation rates on each side of the organ

(6) $$\dot{E}(s,t)=\frac{{\dot{\epsilon }}_{1}(s,t)+{\dot{\epsilon }}_{2}(s,t)}{2}$$

and Δ(*s, t*) is a dimensionless differential growth term which expresses the amount of growth distributed to the differential growth

(7) $$\Delta (s,t)=\frac{{\dot{\epsilon }}_{2}(s,t)-{\dot{\epsilon }}_{1}(s,t)}{{\dot{\epsilon }}_{1}(s,t)+{\dot{\epsilon }}_{2}(s,t)}$$

It is important to note that *Ė*(*s, t*) and Δ(*s, t*) can be defined independently. If there is no shrinkage of the organ, $\dot{\epsilon }$_1_(*s, t*) ≥ 0 and $\dot{\epsilon }$_2_(*s, t*) ≥ 0, then |Δ| ≤ 1. The characteristic length can be given by the radius of the organ *R*. By the same token, the characteristic time can be given by the inverse of the relative elongation rate of the median line *Ė*(*s, t*)^−1^, which gives the time for doubling the length [at constant *Ė*(*s, t*)]. As long as the radius of curvature *C*(*s, t*)^−1^ remains large compared to the radius *R* of the organ, i.e., *CR* ≪ 1, the quadratic prefactor in Equation (5) can be neglected, so the equation can be simplified

(8) $$\frac{DC(s,t)R}{Dt}\sim \dot{E}(s,t)\Delta (s,t)$$

**Figure 3:**
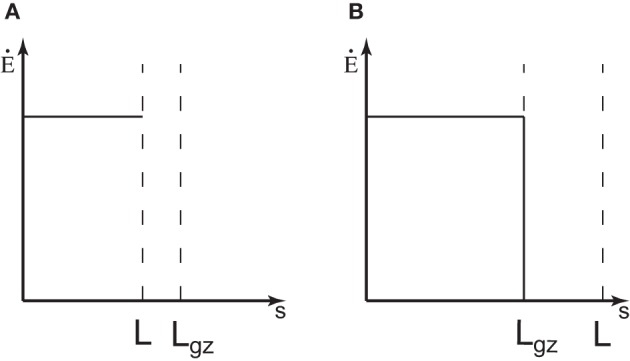
**The elongation rate of an organ. (A)** When the organ is shorter than the length of the growth zone, *L* < *L*_*gz*_, it is expected that the whole organ is elongating. For simplicity, it is considered that the elongation rate is constant along the organ *Ė*_0_. **(B)** If the length of the organ is longer than the elongation zone, *L* > *L*_*gz*_, the elongation rate is equal to 0 outside the growth zone.

Equation (8) expresses how the rate of change in curvature is produced by the combination of the mean elongation rate and the relative amount of growth distributed to the differential growth. It thus expresses the motor role of elongation growth in the tropic movement. There is another element of growth that should be taken into account when considering tropic reactions. In many linear organs, like hypocotyls or primary roots, elongation is limited to a zone just below the apex (Figure 1) (Silk, 1984; Gendreau et al., 1997; van der Weele et al., 2003). All the points at a distance greater than *L*_*gz*_ have stopped growing, *L*(*t*) − *s* > *L*_*gz*_, where *L*(*t*) is the total length of the organ from the apex at time *t*. As growth is the motor of curvature variation (see Equation 8), only the elements inside the elongation zone are able to undergo tropic bending. When a material element has been pushed out of the elongation zone and ceases elongation, *Ė*(*s* > *L*(*t*) − *L*_*gz*_, *t*) = 0, its curvature cannot be modified any more (see Figure 1 in Moulia and Fournier, 2009). In most real cases *Ė*(*s, t*) displays a skewed bell curve, but as a simple starting point, the distribution of *Ė*(*s, t*) can be assumed to be a discontinuous step function that is stationary in time *Ė*(*s* < *L*(*t*) − *L*_*gz*_, *t*) = *E*_0_ (Figure 3). This simplification is expected to capture the main feature of the growth zone, that it first increases in size, *L*(*t*), and then has a constant length, *L*_*gz*_. The curvature variation is thus defined at every position *s* and at any time *t* as

(9) $$s<L(t)-L_{gz}\;\frac{DC(s,t)R}{Dt}=0$$

(10) $$s>L(t)-L_{gz}\;\frac{DC(s,t)R}{Dt}={\dot{E}}_{0}\Delta (s,t)$$

So as the length of the organ *L*(*t*) increases over time, the length of the portion of the organ which can no longer curve, *L*_*f*_(*t*), increases too. Another factor to consider is that there are different phases of growth during organ development. In the first phase, when the organ is very young it elongates along its entire length. The length increases exponentially during this “exponential growth phase.” We can define the length of the growth zone as *L*_*gz*_. Some tissues then start to differentiate and cease extension. In this second phase, when (*L* > *L*_*gz*_), only the subapical part of the organ is elongating, the length of growth zone *L*_*gz*_ is almost constant, and the growth rate remains steady and maximal. The length of the whole organ increases approximately linearly with time during this “linear growth phase.” In a third phase, which will not been studied here, the growth rate and the length of the growth zone decline until growth stops completely.

### Control of differential growth by graviception

How does perception drive movement by modulating differential growth? When there is no differential growth, when Δ = 0, curvature can not be modified even if the organ is elongating, *Ė* > 0 (see Equation 8). Furthermore, as there is no shrinkage of the organ, for a given Δ the relative elongation rate of the median can not modify the sign of the curvature variation, i.e., its direction. Therefore we can assume that the perception affects Δ(*s, t*) and not *Ė*(*s, t*). Note that this is consistent with molecular studies of the distribution of the plant hormone auxin, which enhances cell elongation during the tropic response (Philippar et al., 1999). The total flux of auxin drives the mean elongation rate *Ė* while its transverse redistribution in tropisms drives the relative differential growth (Silk, 1984). Keeping the graviproprioceptive equation of the *AC* model, but assuming that it only drives the distribution of differential growth Δ(*s, t*), yields

(11) $$\Delta (s,t)=-\tilde{\beta }A(s,t)-\tilde{\gamma }C(s,t)R$$

The tilde symbols on top of the parameters β and γ in Equation (11) is to indicate that the dimensions of these sensitivities have changed compared to the “non-growing” *AC* model (Equation 2) (Bastien et al., 2013) as the terms for differential growth in Equation (8) and 11 were broken down. The graviceptive sensitivity $\tilde{\beta }$ and the proprioceptive sensitivity $\tilde{\gamma }$ are now both dimensionless so that $\tilde{\beta }$ = β*R*/*Ė* and $\tilde{\gamma }$ = γ/*Ė*. By doing this, it is now possible to directly compare the graviceptive and proprioceptive parameters of two different plants, independently of their size and mean elongation rate. The dimensionless number *B* can be expressed as:

(12) $$B=\frac{\tilde{\beta }L_{gz}}{\tilde{\gamma }R}$$

Finally, from Equations (8, 11), the *ACĖ* model is defined by

(13) $$\frac{DC(s,t)R}{Dt}=\dot{E}(s,t)\left(-\tilde{\beta }A(s,t)-\tilde{\gamma }C(s,t)R\right)$$

Note that two plants with the same diameter *R* and the same sensitivities $\tilde{\beta }$ and $\tilde{\gamma }$ can nevertheless behave differently at a given time *t*. For example, if the plants are elongating at different rates, the plant with the higher growth rate will converge to the vertical first.

### Sources of experimental data

The experimental data used to assess the *ACĖ* model are those described in the study from Bastien et al. (2013). Experiments on etiolated wheat coleoptiles (*Triticum aestivum* cv. *Recital*) were conducted in growth cabinets. Experiments on bean hypocotyls (*Phaseolus vulgaris*), sunflower hypocotyls (*Helianthus annuus*), pea epicotyls (*Pisum sativum*), tomato stems (*Solanum lycopersicum*), chili stems (*Capsicum annuum*), raspberry canes (*Rubus ideaus*), carnation inflorescences (*Dianthus caryophyllus*) and *Arabidopsis thaliana* inflorescences (ecotype Col0) were conducted in controlled temperature greenhouses. Plants were grown until a chosen developmental stage of the organ of interest (e.g., until the beginning of inflorescence flowering for Arabidopsis). They were then tilted and clamped so the organ to be observed was horizontal so *A*(*s* = 0, *t*) = *A*_0_ = π/2 for all *t* under constant environmental conditions in the dark (to prevent phototropism). The numbers of replicates were 30 for wheat, 15 for Arabidopsis, and 5 for all the other species. Data from similar experiments on *Impatiens glandilufera* stems by Pfeffer (Bastien et al., 2013) and on poplar trunks (*Populus deltoides x nigra cv I4551*) by Coutand et al. (2007) were also reprocessed and used. Simplified morphometric characteristics were measured following the methods described in Bastien et al. (2013). To take into account the two types of growth distribution (Figure 3), an effective length *L*_*eff*_ is defined as *L*_*gz*_ when *L* > *L*_*gz*_ and the initial length of the organ *L*_0_ when *L*_*gz*_ < *L*. The effective length *L*_*eff*_ was estimated by superimposing the first and last kinematics images. The effective length of the organ is defined as the distance from the point where the plant starts to curve to the apex of the plant in the first image. The convergence length *L*_*c*_ was obtained by plotting the local inclination angle *A*(*s*) along the organ beginning from the curved zone at the steady state. The angle *A*(*s*) was then fitted with the exponential *A*(*s*) = *A*_0_*e*^−*s/L*_*c*_^.

Here we first analyze the destabilizing effects of growth on the tropic movements and on the final shape of the organ, namely fixed curvature and passive orientation drift. We then analyze the behavior of the *ACĖ* model in the two cases of growth distribution along the organ, exponential growth when *L* < *L*_*gz*_ and linear growth when *L* > *L*_*gz*_. Special attention will be paid to the situations in which growth effects may be neglected, when the simpler *AC* model may provide a sufficiently good estimate of the behavior of the more complete but complex *ACĖ* model.

### Fixed curvature

During the primary growth of plant organs, steady linear growth is usually achieved (in the absence of environmental stresses) in the first few days of organ development. Elongation then becomes restricted to a subapical zone whose length is *L*_*gz*_. Values of *L*_*gz*_ and *Ė*_0_ remain almost constant for a few days (Figure 3B) (Silk, 1984). At this stage, the organ is only able to modify its own curvature from within the subapical growth zone (e.g., *s* > *L*(*t*) − *L*_*gz*_). The length of fixed matured tissues convected out of the growth zone is given by

(14) $$L_{f}(t)=L(t)-L_{gz}$$

If a material element that is convected out of the growth zone is curved, its curvature can no longer be modified and will remain a fixed part of the final shape.

### Passive orientation drift

**Figure 4:**
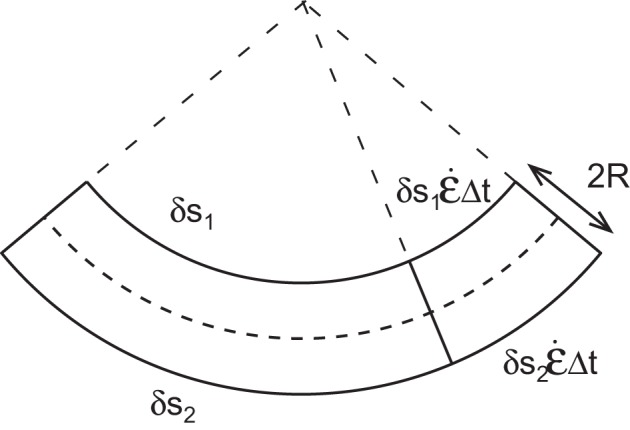
**An element of a curved organ is elongating with the same growth rate $\dot{\epsilon }$ on each side**. The increment of length does not affect the curvature of the organ but the final orientation of the element is changed.

When there is longitudinal expansion (*Ė*(*s, t*) > 0) with no differential growth (Δ(*s, t*) = 0) (Figure 4), there is no variation in curvature $\frac{DC}{Dt}=0$. However, as the length of the element can increase due to elongation, the orientation of the two ends of the element might be modified. The difference in orientation *A*′(*s, t*) across a short segment δ*s* is given by *A*′(*s, t*) = *C*(*s, t*)δ*s*. The variation in orientation *dA*′(*s, t*)/*dt* of this segment over time can be expressed as (see Supplementary Material)

(15) $$\frac{dA^{\prime }(s,t)}{dt}={\dot{E}}_{0}(\Delta (s,t)+C(s,t)R)\frac{\delta s}{R}$$

This equation only applies to an independent element of infinitesimal length δ*s*, but not to the whole organ. As the perception and response are local (Bastien et al., 2013), the constraint on the regulation of the shape must take place at the scale of each element independently of the other segments.

When Δ(*s, t*) = 0 and the organ is curved, the orientation between the two ends of a material element is modified proportionally to its curvature. To achieve an active control over the movement, the differential growth term *Ė*(*s, t*)Δ(*s, t*) must be larger than the term for passive orientation drift, *Ė*(*s, t*)*C*(*s, t*)*R*, yielding

(16) |Δ(s,t)| > |C(s,t)R|

(17) $$\left|-\tilde{\beta }A(s,t)-\tilde{\gamma }C(s,t)R|\;>\;|C(s,t)R\right|$$

This process must be independent of the orientation so that the plant remains capable of active control even for small angles of perturbation, i.e., when *A* is close to 0

(18) $$\tilde{\gamma }|C(s,t)R|\;>\;|C(s,t)R|$$

(19) $$\tilde{\gamma }>1\;\;\;\;\;\;\;\;\;\forall A,C$$

For an active tropic control to be possible, the proprioceptive term must overreach the effect of the passive orientation drift. The condition for this in an elongating system reduces simply to the value of the proprioceptive term $\tilde{\gamma }$ being greater than 1. This means that the typical time for convergence to the steady state under the proprioceptive drive *Ė*^−1^_0_γ^−1^ should be smaller than the typical time for expansion growth *Ė*^−1^_0_.

### Exponential growth

**Figure 5:**
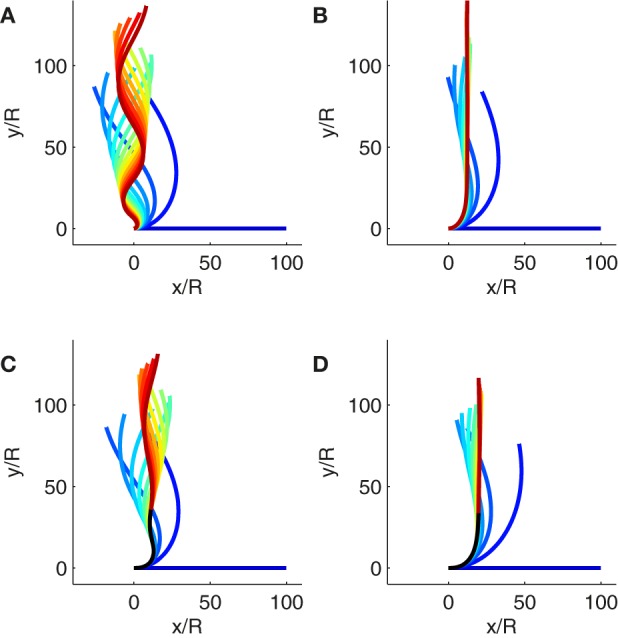
**Simulations of gravitropic growth**. The color (from blue to red) codes for the elapsed simulation time. **(A)** Solution of the *ACĖ* model during exponential growth when *B* = 10, $\tilde{\gamma }$ = 0.1 and *L*_0_/*R* = 100. The simulated organ does not reach a steady state. The size of the curved zone is expanded by growth and the organ cannot regulate its posture (Movie 1). **(B)** Solution of the *ACĖ* model during exponential growth when *B* = 10, $\tilde{\gamma }$ = 10 and *L*_0_/*R* = 100. The simulated organ reaches a steady state even if the organ is elongating (Movie 2). **(C)** Solution of the *ACĖ* model during subapical growth when *B* = 10, $\tilde{\gamma }$ = 0.1, and *L*_*gz*_/*R* = 100. The black curve is the part of the organ that is outside the growth zone where the curvature cannot be modified. As the simulated organ reaches the vertical, oscillations are fixed on the final shape due to the elements convected outside the growth zone (Movie 3). **(D)** Solution of the *ACĖ* model during subapical growth when *B* = 10, $\tilde{\gamma }$ = 10, and *L*_*gz*_/*R* = 100. The black curve is the part of the organ that is outside the growth where the curvature cannot be modified. The simulated organ reaches a steady state before the elements of the organ are convected outside of the growth zone. No oscillations are fixed on the final shape (Movie 4).

We can now consider the kind of growth that is observed in very young organs when the length of the organ increases exponentially over time (Figure 1). In this case the relative elongation rate *Ė*(*s, t*) is homogenous all along the organ and is homogenous in time (*Ė*(*s, t*) = *Ė*_0_ is considered to be constant). As seen previously with passive orientation drift, according to Equation (19) the system cannot converge if $\tilde{\gamma }$ < 1. Spatial oscillations of curvature along the organ are amplified over time. When the orientation of the organ tip reaches the vertical, the organ is still curved, and the effect of passive orientation drift spreads due to continued growth (see Figure 5A and Movie 1). Conversely when $\tilde{\gamma }$ > 1 the system can converge to a steady state (see Figure 5B and Movie 2). To maintain a steady shape despite the continuous disturbance from passive orientation drift due to growth, posture must be constantly regulated. This contrasts with the *AC* model for non-elongating organs where it is sufficient to balance graviception with proprioception.

The steady state of the *ACĖ* model undergoing exponential growth is given by (see Supplementary Material for more details)

(20) $$A(s,t)=A_{0}\Gamma (\tilde{\gamma }){\left(\frac{\tilde{\beta }s}{R}\right)}^{\frac{1-\tilde{\gamma }}{2}}J_{\tilde{\gamma }-1}\left(2\sqrt{\frac{\tilde{\beta }s}{R}}\right)$$

Bessel functions of the first kind *J*_*n*_ are oscillating functions, and Γ(*x*), the gamma function, is an extension of the factorial function *x*! to real numbers. As there are two Bessel functions, the steady state can stabilize some spatial undulations to produce a wavy shape (see Supplementary Material Figure 1). Such oscillations can be avoided only if the first zero of the Bessel function is displaced toward the tip of the organ. One way to do this is to increase the order of the Bessel function, i.e., by increasing *n* = $\tilde{\gamma }$ − 1 in Equation (20) (so the first zero of *J*_*n*_(*x*) is displaced to higher *x* values for increasing values of *n*). Only high $\tilde{\gamma }$ can avoid undulations in the steady state. As found previously for the control of passive orientation drift, the value of the proprioceptive sensitivity is central to avoiding undulations in the steady state shape, and hence to achieving proper gravitropism (Bastien et al., 2013).

### Conditions for neglecting exponential growth

When does the simple *AC* model provide a good approximation for the behavior of the more complete but complex *ACĖ* model for exponential growth? (See Supplementary Material for a mathematical justification of the approximation of the steady states of the two models).

We need to define “good approximation.” As the *AC* and *ACĖ* models are intended to explain experimental results, a good approximation will be one where the difference between the *AC* and the *ACĖ* models is smaller than the experimental error in the “best” experiments. In our experiments the error in measurement of the orientation of the organ is of the order of 0.05 radians (Bastien et al., 2013). If the absolute difference between the steady state of the *ACĖ* model (Equation 20) and the steady state of the *AC* model (*A*(*s*) = *A*_0_*e*^−*Bs/L*_*gz*_^), called μ,

(21) $$\mu =\left|A_{0}\Gamma (\tilde{\gamma }){\left(\frac{B\tilde{\gamma }s}{L_{gz}}\right)}^{\frac{1-\tilde{\gamma }}{2}}J_{\tilde{\gamma }-1}\left(2\sqrt{\frac{B\tilde{\gamma }s}{L_{gz}}}\right)-A_{0}e^{-Bs/L_{gz}}\right|$$

is less than 0.05, it is not possible to distinguish between the two models. Numerically μ ≤ 0.05 if $\tilde{\gamma }$ > 6.2 (Figure 2 Supplementary Material). This approximate limit must, however, be expressed with measurable parameters from experiments if we are to test the validity of the approximation. As stated in the introduction, it is known that initiation of gravitropism in an organ results in an inhibition of growth on one side of the organ with a corresponding increase on the other side (Cosgrove, 1990; Myers et al., 1995; Tokiwa et al., 2006). According to Equation (7), this leads to |Δ| ~ 1. According to Equation (11), at the beginning of the tropic response, the distribution of differential growth is dominated by graviception so |Δ| ~ β|*A*_0_| ~ 1. However, this expression is only valid for small angles. Gravisensing at any angle was shown to follow a sine law (see Equation 1). Following the same argument as previously, it can be shown that |Δ| ~ β| sin *A*| ~ 1. As | sin *A*| ≤ 1, this yields β ~ 1.

With $\tilde{\beta }$ ~ 1 and Equation (12), a simple relationship between the proprioceptive term $\tilde{\gamma }$, the length of the growth zone *L*_*gz*_ and *B* can be stated as

(22) $$\tilde{\gamma }=\frac{L_{gz}}{BR}$$

The approximate limit defined for $\tilde{\gamma }$ can now be rewritten using measurable parameters *B* and Ł_*gz*_ (Bastien et al., 2013)

(23) $$\frac{L_{gz}}{R}>6.2B$$

Equation (23) thus describes a condition for which the *AC* model gives an approximation of the steady state of the *ACĖ* model that lies within the range of experimental error.

### Subapical elongation and linear growth

Growth is exponential only at the very start of organ development. Later the length of the growth zone is always finite, a generic feature of plant growth. Even in tiny coleoptiles, the length of the coleoptile soon overreaches *L*_*gz*_, and non-growing tissues accumulate at the base (Silk et al., 1989; Peters and Tomos, 2000; Walter et al., 2002). As the elements are convected out of the growth zone, the plant loses the ability to modify its curvature. Some plants overshoot the vertical during the gravitropic movement and display transient spatial oscillations (Bastien et al., 2013). If elements are convected outside of the growth zone while overshooting the vertical, spatial oscillations could remain fixed on the final shape. Due to the complexity of the *ACĖ* model in this case, the steady state shape cannot be worked out analytically. However, the behavior of the model can be studied by numerical simulations. Figures 5C,D and Movies 3 and 4 show how the final steady-state shape (including fixed curvature) may vary depending on whether or not transient curvature becomes fixed. As all of the plants studied experimentally so far exhibited smooth convergence to the vertical without fixed oscillations of curvature (Bastien et al., 2013), it is important to clarify the conditions under which curvature undulation does not become fixed in the *ACĖ* model as a way of checking the validity of the modeling.

### Conditions to avoid the fixation of curvature

If the organ has time to converge to a steady state before a vertical element is convected out of the growth zone, no spatial oscillations are fixed on the final shape as there will be no inflection point within the zone of fixed curvature. To assess whether spatial oscillations are fixed into the final shape, the time for a vertical element to be convected out of the growth zone, *T*_*f*_, has to be compared with the time to converge to the steady state, *T*_*c*_. If *T*_*c*_ ≫ *T*_*f*_, then a vertical element is convected outside of the growth zone while the organ is still regulating its posture. The organ cannot avoid fixation of transient over-curvatures during the overshoots of the vertical. However, if *T*_*c*_ ≪ *T*_*f*_, when a vertical element is convected outside of the growth zone, the organ has already converged to its steady state, and this non-undulating steady-state shape is then fixed.

The convergence time can be approximated from the proprioceptive term that dominates when approaching convergence

(24) $$T_{c}=\frac{1}{\dot{E}\tilde{\gamma }}$$

To express *T*_*f*_, it is necessary to know the length of the elements of the organ that have left the elongation zone and lost their ability to curve. After time *t* the length of the organ is

(25) $$L(t)=L_{gz}(1+\dot{E}t)$$

By Equation (14), the length of fixed “mature” tissues convected out of the growth zone is given by

(26) $$L_{f}(t)=L(t)-L_{gz}=L_{gz}{\dot{E}}_{0}t$$

To estimate the time required to fix a vertical element, we can consider an extreme case. If the whole plant is curving at the same rate all along the organ, depending only on the graviceptive term at the initial angle *A*_0_, it will have a maximal bending rate. Assuming that this maximal rate is kept constant until the tip first reaches the vertical, a lower limit of the real time to reach the vertical can be estimated and should give the most stringent condition for neglecting fixed curvature. Thus the curvature of an element that leaves the growth zone at time *t* is given by

(27) $$C(L_{f}(t),t)R=-\tilde{\beta }A_{0}{\dot{E}}_{0}t$$

Integration over the fixed elements gives the angle reached by an element outside the growth zone

(28) $$A(L_{f}(t),t)=A_{0}-\tilde{\beta }A_{0}{\dot{E}}_{0}t\frac{L_{f}(t)}{R}$$

The first vertical element outside the growth zone is given simply by *A*(*L*_*f*_(*T*_*f*_), *T*_*f*_) = 0. From Equations (28, 26), the minimal time for a vertical element to leave the growth zone is given by

(29) $$T_{f}={\dot{E}}^{-1}\sqrt{\frac{2R}{\tilde{\beta }L_{gz}}}$$

The ratio between the time to converge to the steady state and the time to fix a vertical element, $\frac{T_{c}}{T_{f}}=\frac{1}{\tilde{\gamma }}\sqrt{\frac{\tilde{\beta }L_{zc}}{2R}}$, should be as low as possible for straightening to start and a straight vertical shape to be established. This defines a condition for proper straightening, $\tilde{\gamma }\gg \sqrt{\frac{\tilde{\beta }L_{zc}}{2R}}$, and the higher the value the better the straightening. Equation (22) expresses this approximate limit as a function of parameters that can be measured experimentally, *B* and *L*_*gz*_ where

(30) $$B<\sqrt{\frac{2L_{gz}}{R}}$$

The dynamics of the organ is then confined close to the dynamics of the non-elongating model (with negligible curvature fixed in the mature basal zone). However, sub-apical growth restrains the range of values for the parameter $\tilde{\gamma }$ that make such straightening behavior possible.

### Experimental validation

**Figure 6:**
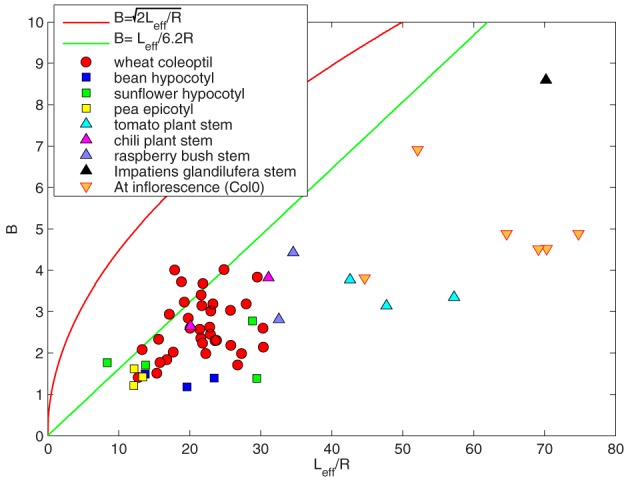
***B* = *L*_*eff*_/*L*_*c*_ as a function of the effective length of the organ *L*_*eff*_/*R***. Each point denotes an individual plant. The red line represents $B=\sqrt{L_{eff}/R}$. All the points are under this line $B<\sqrt{L_{eff}/R}$, showing that the destabilizing effects of growth on the whole gravitropic movement are negligible. The green line accounts for *B* = *L*_*eff*_/6.2*R*. Most of the points are under this line, showing that it is not possible to distinguish the steady state of the *ACĖ* model from the *AC* model.

The value of *B* was found experimentally to range from 0 to 10, with most of the observed values ranging from 2 to 5 (Figure 6). Results in Figure 6 show that most of the plants tested fulfilled the conditions in which passive orientation drift can be neglected (Equation 23). Therefore in the range of experimental values of B, it is not possible to distinguish between the *ACĖ* and the *AC* models. It follows that the solution of the *AC* model without elongation is a good approximation of the *ACĖ* model. However, the growth of some wheat coleoptiles did not fulfil these conditions (Equation 23). It has already been noted that these coleoptiles oscillated less during the gravitropic movement than predicted by the *AC* model (Bastien et al., 2013). This behavior cannot be accounted for using the *ACĖ* model either, as the effects of growth identified in this paper are destabilizing effects that do not reduce oscillation. It is then likely that the effects of growth are negligible in the wheat coleoptile and that second-order mechanisms of regulation are involved. Overall the *AC* model that does not explicitly include elongation (Bastien et al., 2013) remains an excellent first-order approximation for an elongating organ. This is despite the effects of passive orientation drift and the fact that the observed final shapes in real plants are in fact dynamical steady states in which the organ is continuously expanding (described more precisely in the *ACĖ* model).

The effects of fixed curvature can be neglected if B is in good agreement with the approximate bounds given by Equation (30). This condition can be experimentally tested by comparing *L*_*gz*_ with *B* (Figure 6). All the plants of the different species and in all experiments were found to match the approximate lower limit given by relationship 30. Even if the length of the organ *L* is greater than the length of the growth zone *L*_*gz*_, the *AC* model is still a good approximation of the *ACĖ* model.

## Discussion-conclusion

The *ACĖ* model developed here is an attempt to combine two branches of knowledge about gravitropism. Is it possible to describe the role of differential growth mediated by enhanced lateral auxin transport (Silk, 1984; Moulia and Fournier, 2009; Morita, 2010) in the context of the combined control of graviception and proprioception formalized in the *AC* model (Bastien et al., 2013)?

The *ACĖ* model was built by extending the *AC* model with a simplified kinematic equation expressing how growth is the motor for the curvature variation in gravitropism (Equation 8). This equation directly relates the rate of curvature variation attached to a material element (an infinitesimal segment of the organ) $\frac{DC}{Dt}$ to the mean elongation *Ė* and the relative differential growth rate Δ between each side of the organ. As in the *AC* model, the perception process combining graviception and proprioception drives the movement, but it is now assumed that this motor acts by controling the differential growth rate Δ (while the mean elongation rate remains constant). Introducing the motor of curvature variation in the model (Equation 8) has helped to identify two main effects of growth on the gravitropic response of shoots, and their consequences on achieving proper control over the tropic movement.

First, the modification of the curvature can only occur when the relative differential growth is not equal to zero Δ ≠ 0. Despite appearing trivial, this result contradicts the classical view of growth. It might be intuitively accepted that elongation of a curved cylindrical organ, without differential growth, decreases the curvature of this organ. Statements of this kind of intuition can be traced back at least as far as (Jost and Gibson, 1907), but the kinematics have not been demonstrated before. Here we show that if a material element is elongating with no differential growth, the curvature of this element is conserved. But the size of this element does increase. Therefore the orientation of its distal end, resulting from the integral of the curvature over the length of the element, is modified, due to curvature spreading over elongation growth. This effect has been called “passive orientation drift.” The consequence of such passive orientation drift is that the orientation of the growing curved organ must be regulated all the time for tropic control to be effective. Elongating organs can thus only achieve a purely dynamical tropic steady-state shape through a dynamical balance between the passive orientation drift and the active differential growth driven by graviproprioceptive control.

Even when the shape remains stationary, the material elements are “flowing” and their shapes must be actively regulated at all times. This is a new example of the maintenance of “steady shape from changing cells” (Silk and Erickson, 1978) which has been primarily described for the maintenance of the hook of some hypocotyls during their etiolation. More precisely, we have shown that to overcome passive orientation drift and reach a tropic steady-state shape, an approximate limit can be defined that relates the length of the growth zone *L*_*gz*_ and its radius *R* to the bending number *B* where *B* represents the driving of the motion through the combined action of graviception and proprioception all along the growth zone. Thus only organs with proper sets of values for the growth zone aspect ratio, *L*_*gz*_/*R*, and for the bending number *B* can control posture and convergent tropic movement. For example, organs with a high value for the bending number *B* will only reach a tropic steady state if they also have a long growth zone, *L*_*gz*_.

The second effect of growth on the gravitropic response of shoots has been called the “fixed curvature.” Again, this starts from an apparently trivial observation. All the elements outside the growth zone are no longer able to actively change their curvature or orientation [at least until secondary cambial growth begins (see Moulia and Fournier, 2009)]. But the consequence is not trivial and the *ACĖ* model is needed to elucidate this aspect of tropic growth. We showed that the organ must converge to the steady state shape fast enough so that only a negligible amount of transient curvatures remains fixed at the base. Experiments have shown that plants mostly exhibit a monotonic decrease in orientation from the tilted base to the almost vertical apex (Bastien et al., 2013). This is a good indicator that no curvature has become fixed. To avoid the fixing of transient curvatures, a new approximate limit has been defined that relates the length of the growth zone, *L*_*gz*_, and the bending number *B* and thus the ratio between the graviceptive and the proprioceptive sensitivities.

By revisiting the experimental data on gravitropic movements from several very different species and over two order of magnitude in organ size, we have shown that all these plant movements are within the approximate bounds we defined by the modeling. In other words, all the species and organ types studied so far have avoided fixed curvature and overcome passive orientation drift (Bastien et al., 2013). Although gravitropism is thought to be adaptive (Moulia et al., 2006), there is very little evidence to support this. Our results may be the first evidence of converging selection for phenotypic sets of (*L*_*gz*_, *R, B*) values that bring about efficient posture control. More direct studies of population genetics would be a way of investigating this hypothesis.

The approximation of the *ACĖ* model with the *AC* model does not mean that growth can always be neglected. The timing of the tropic movement is still fixed primarily by the mean relative rate of elongation growth. There are other aspects that could be included in modeling, especially the mechanical aspects powering the motor of differential growth (Moulia and Fournier, 2009) or conditioning the mechanical stability of the straightened and erected shape. The tropic movement of the plant organs with secondary growth in girth such as tree trunks and branches remains also to be studied. The motor for the curvature change in this case is not differential growth but differential shrinkages or expansion in the wood (Alméras and Fournier, 2009; Moulia and Fournier, 2009).

The fact that plants were shown to fulfil the conditions required to overcome the two destabilizing effects of growth on plant movement has an overriding practical aspect. In these conditions, the original *AC* model is a good approximation for the more general but complex *ACĖ* model. This means that gravitropism and posture control can be characterized phenotypically by the measuring the dimensionless parameter *B* based only on the *AC* model.

### Conflict of interest statement

The authors declare that the research was conducted in the absence of any commercial or financial relationships that could be construed as a potential conflict of interest.
