# Supplementary material for: A unifying modeling of plant shoot gravitropism with an explicit account of the effects of growth
Source: Front Plant Sci. 2014 Apr 14;5:136. doi: 10.3389/fpls.2014.00136 (PMC3995075; doi:10.3389/fpls.2014.00136)
Supplement: Supplementary file 1 [file Presentation1.PDF]

CURVATURE VARIATION

There is first a geometrical relation between the curvature and the length of each side, δs_1 and δs_2

$$CR = \frac{\delta s_1 - \delta s_2}{\delta s_1 + \delta s_2} \tag{1}$$

After a time dt, each side has been elongating. The new size of the element is then defined as

$$\delta s_1 \rightarrow \delta s_1 + \dot{\epsilon}_1 \delta s_1 dt \tag{2}$$
$$\delta s_2 \rightarrow \delta s_2 + \dot{\epsilon}_2 \delta s_2 dt \tag{3}$$

while the curvature is modified

$$C \rightarrow C + dC \tag{4}$$

Replacing the equations 2, 3 and 4 inside the equation 1, yields the temporal evolution of the curvature of a material element with differential growth rate as

$$\frac{R}{2}\frac{dC}{dt} = (1 - C^2R^2)(\dot{\epsilon}_1 - \dot{\epsilon}_2) \tag{5}$$

with s the curvilinear abscissa defined from the base, $s = 0$, to the apex, $s = L$. In the expanding organs however, the successive segments are moving along the organ relative to each other. It is then necessary to take into account the material derivative comoving with each element of organ (see Silk 1984 and Moulia and Fournier 2009 for more details). In the referential defined by the curvilinear abscissa along the median line of the organ from the base to the apex (Figure 1), this material derivative is defined by

$$\frac{DC(s,t)}{Dt} = \frac{\partial C(s,t)}{\partial t} + v(s,t)\frac{\partial C(s,t)}{\partial s} \tag{6}$$

where $v(s,t)$ is the velocity of the growth-induced displacement of each element along the organ. Equation 5 is rewritten as

$$\frac{DC(s,t)}{Dt} = \frac{1}{2R}\left(1 - C(s,t)^2R^2\right)\left(\dot{\epsilon}_2(s,t) - \dot{\epsilon}_1(s,t)\right) \tag{7}$$

If the limit of small curvature, $C(s,t)R \ll 1$, this equation becomes

$$\frac{DC(s,t)}{Dt} \sim \frac{1}{2R}\left(\dot{\epsilon}_2(s,t) - \dot{\epsilon}_1(s,t)\right) \tag{8}$$

Silk (1984) defines a different equation

$$\frac{\partial}{\partial t}\log\left(1 + K(s,t)w\right) + v(s,t)\frac{\partial}{\partial s}\log\left(1 + K(s,t)R\right) = \dot{\epsilon}_2(s,t) - \dot{\epsilon}_1(s,t) \tag{9}$$

where $w = 2R$ is the width of the organ and $K(s,t)$ is the curvature of a side of the organ. With the parameters defined in this paper, and taking into account the curvature on the median line, this equation

can be expressed as

$$\frac{\partial}{\partial t}\log\left(\frac{1+C(s,t)R}{1-C(s,t)R}\right)+v(s,t)\frac{\partial}{\partial s}\log\left(\frac{1+C(s,t)R}{1-C(s,t)R}\right)=\dot{\epsilon}_2(s,t)-\dot{\epsilon}_1(s,t) \tag{10}$$

First, the derivative $\frac{\partial}{\partial t}\log\left((1+C(s,t)R)/(1-C(s,t)R)\right)$ is expanded

$$\frac{\partial}{\partial t}\log\left(\frac{1+C(s,t)R}{1-C(s,t)R}\right)=\frac{1+C(s,t)R}{1-C(s,t)R}\frac{(1-C(s,t)R)+(1+C(s,t)R)}{(1-C(s,t)R)^2}\frac{\partial C(s,t)}{\partial t}R \tag{11}$$

yielding

$$\frac{\partial}{\partial t}\log\left(\frac{1+C(s,t)R}{1-C(s,t)R}\right)=\frac{2}{1-C(s,t)^2R^2}\frac{\partial C(s,t)}{\partial t}R \tag{12}$$

The same expression can easily be obtained for $\frac{\partial}{\partial t}\log\left((1+C(s,t)R)/(1-C(s,t)R)\right)$. Thus, from equation 10, one can write

$$\frac{2}{1-C(s,t)^2R^2}\left(\frac{\partial C(s,t)}{\partial t}R+v(s,t)\frac{\partial C(s,t)}{\partial s}R\right)=(\dot{\epsilon}_2(s,t)-\dot{\epsilon}_1(s,t)) \tag{13}$$

and the full solution is

$$\frac{DC(s,t)}{Dt}=\frac{1}{2R}(1-C(s,t)^2R^2)(\dot{\epsilon}_2(s,t)-\dot{\epsilon}_1(s,t)) \tag{14}$$

which is strictly equivalent to the equation 7.

From the equation 4 and 10 of Chavarria-Krauser 2006 it is possible to define a third equation for the curvature variation of a growing organ. It can be expressed with the parameters defined in this paper as

$$\frac{DC(s,t)}{Dt}=\frac{\dot{\epsilon}_2(s,t)-\dot{\epsilon}_1(s,t)}{2R}+C\left(\frac{\dot{\epsilon}_2(s,t)+\dot{\epsilon}_1(s,t)}{2}-\dot{E}\right) \tag{15}$$

where $\dot{E}$ is the elongation rate of the median line. In the limit of small curvature $\dot{E}\sim\frac{\dot{\epsilon}_2(s,t)+\dot{\epsilon}_1(s,t)}{2}$, yielding

$$\frac{DC(s,t)}{Dt}\sim\frac{\dot{\epsilon}_2(s,t)-\dot{\epsilon}_1(s,t)}{2R} \tag{16}$$

which is equivalent to the equation we defined in the limit of smal curvature (equation 8).

ORIENTATION OF AN ISOLATED ELEMENT

The difference of orientation $A'(s,t)$ across a segment of small length δs is given by

$$A'(s,t)=A(s+\delta s,t)-A(s,t)=C(s,t)\delta s \tag{17}$$

The material element is elongationg with a median elongation rate $\dot{E}_0$. After a time dt, the orientation is modified as

$$A'(s,t)+dA'(s,t)=(C(s,t)+dC(s,t))\delta_s(1+\dot{E}_0dt) \tag{18}$$

At first order this yields

$$\frac{dA'(s,t)}{dt}=\left(C(s,t)\dot{E}_0+\frac{dC(s,t)}{dt}\right)\delta_s \tag{19}$$

With $\frac{dC(s,t)}{dt}R = \dot{E}_0\Delta(s,t)$

$$\frac{dA'(s,t)}{dt} = \dot{E}_0\left(C(s,t)R + \Delta(s,t)\right)\frac{\delta_s}{R} \tag{20}$$

STEADY STATE DURING EXPONENTIAL GROWTH

The steady state shape of the organ is given by

$$\frac{\partial C(s,t)}{\partial t} = 0 \tag{21}$$

with

$$C(s,t) = \frac{\partial A(s,t)}{\partial s} \tag{22}$$

$$A(s,t) = A_0 + \int_0^s C(l,t)dl \tag{23}$$

In the exponential growth case, the median elongation rate is expected to be constant $\dot{E}(s,t) = \dot{E}_0$. The velocity of a material element along the organ at position s is given by

$$v(s,t) = \int_0^s dl\dot{E}(l,t) = \dot{E}_0 s \tag{24}$$

then equation 21 can be combined with equation 6 yielding

$$\frac{\partial C(s,t)}{\partial t} = \frac{DC(s,t)}{Dt} - \dot{E}_0 s\frac{\partial C(s,t)}{\partial s} \tag{25}$$

Using $\Delta(s,t) = -\tilde{\beta}A(s,t) - \tilde{\gamma}C(s,t)$, equation25 becomes

$$\frac{\partial C(s,t)}{\partial t} = \dot{E}_0(-\frac{\tilde{\beta}}{R}A(s,t) - \tilde{\gamma}C(s,t) - s\frac{\partial C(s,t)}{\partial s}) \tag{26}$$

The steady state is then given by $\frac{\partial C(s,t)}{\partial t} = 0$ yielding to

$$A(s,t) = s^{\frac{1-\tilde{\gamma}}{2}}\left(C_1 J_{\tilde{\gamma}-1}\left(2\sqrt{\frac{\tilde{\beta}s}{R}}\right) + C_2 Y_{\tilde{\gamma}-1}\left(2\sqrt{\frac{\tilde{\beta}s}{R}}\right)\right) \tag{27}$$

where the functions J_n are the Bessel function of the first kind and Y_n the Bessel function of the second kind. The orientation converge to the vertical if $\frac{1-\tilde{\gamma}}{2} < 0$ which implies

$$\tilde{\gamma} > 1 \tag{28}$$

So the condition to overcome the passive growth-induced curvature is sufficient for the steady state of the $AC\dot{E}$ model to have it apical segment lying along the vertical direction.

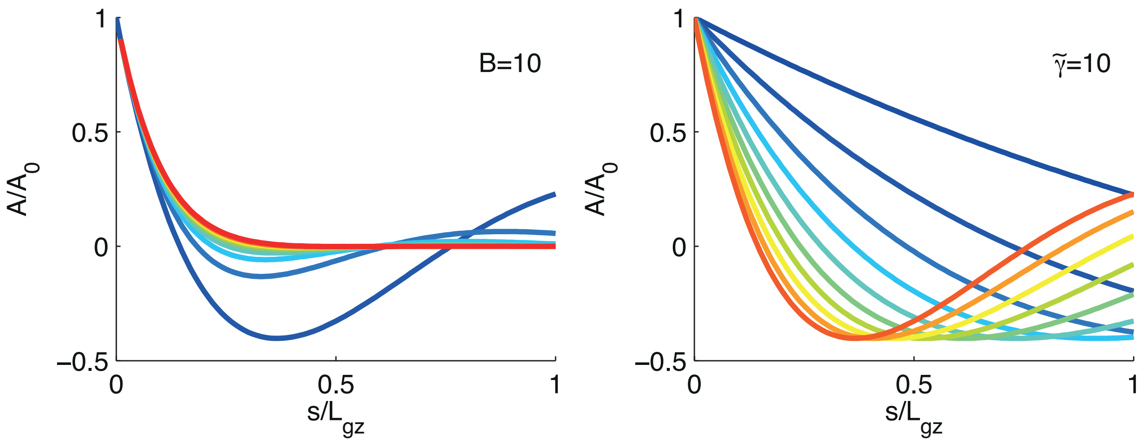

Figure 1. Steady State of $AC\dot{E}$ model, equation 29. A. $B = 10$, as $\tilde{\gamma}$ increases (plain color line from blue to red), the spatial oscillations along the organ are reduced and the function converges smoothly to the vertical ($A = 0$). B. $\tilde{\gamma} = 1$, as B increases (plain color line from blue to red), the organ overshoots the vertical ($A = 0$) and the spatial oscillations along the organ are increased.

The initial condition $A(0,t) = A_0$ allows to identify C_1 and C_2. As $s^{\frac{1-\tilde{\gamma}}{2}}$ and $Y_n\left(2\sqrt{\frac{\tilde{\beta}s}{R}}\right)$ diverges when $s = 0$, then $C_2 = 0$. $s^{\frac{1-\tilde{\gamma}}{2}} J_n\left(2\sqrt{\frac{\tilde{\beta}s}{R}}\right) = \frac{1}{\Gamma(\tilde{\gamma})}\left(\frac{\tilde{\beta}}{R}\right)^{\frac{\tilde{\gamma}-1}{2}}$ when s goes to 0, so it comes directly that $C_2 = A_0\Gamma(\tilde{\gamma})\left(\frac{\tilde{\beta}}{R}\right)^{\frac{1-\tilde{\gamma}}{2}}$.

The steady state is finally given by (Figure 1)

$$A(s,t) = A_0\Gamma(\tilde{\gamma})\left(\frac{\tilde{\beta}s}{R}\right)^{\frac{1-\tilde{\gamma}}{2}} J_{\tilde{\gamma}-1}\left(2\sqrt{\frac{\tilde{\beta}s}{R}}\right) \tag{29}$$

With the development of the Bessel function as a power series, it is possible to write the equation 29 as

$$A(s,t) = A_0\sum_{p=0}^{\infty}\frac{(-1)^p}{p!}\left(\frac{\tilde{\beta}s}{\gamma R}\right)^p\frac{\tilde{\gamma}^p\Gamma(\tilde{\gamma})}{\Gamma(\tilde{\gamma}+p)} \tag{30}$$

The steady state shape of the non growing case is described by

$$A(s,t) = A_0 e^{-\tilde{\beta}s/\tilde{\gamma}R} = A_0\sum_{p=0}^{\infty}\frac{(-1)^p}{p!}\left(\frac{\tilde{\beta}s}{\tilde{\gamma}R}\right)^p \tag{31}$$

So if $\frac{\tilde{\gamma}^p\Gamma(\tilde{\gamma})}{\Gamma(\tilde{\gamma}+p)} \to 1$ is satisfied, the steady state of the $AC\dot{E}$ model, is equivalent to the steady state of the AC model. The conditions where $\Gamma(\tilde{\gamma}+p) \sim \tilde{\gamma}^p\Gamma(\tilde{\gamma})$ is true must be identified. For a finite p, this approximation is valid, if $\tilde{\gamma}$ tends to infinity. In this case the term $\frac{\tilde{\gamma}^p\Gamma(\tilde{\gamma})}{\Gamma(\tilde{\gamma}+p)}$ tends to 1 and there is an equivalence between the the $AC\dot{E}$ model and the AC model. The validity of this approximation must be

discussed more carefully. In fact, this approximation is valid as long as $\tilde{\gamma} \gg p$ but as p could be infinite along the sum, the condition seems unfulfilled for large values of p. However as the series (30) and (31) are convergent, the contributions of the higher order are negligible.

A numerical study of this problem should then allow to define a cut-off for p, p_c can then be defined as function of $\tilde{\beta}$, $\tilde{\gamma}$ and R such as: (i) the contributions of all the terms for $p > p_c$ are negligible in the sum 30 and (ii) the sum 30 up to the term $p = p_c$ is a good approximation of the sum 31.

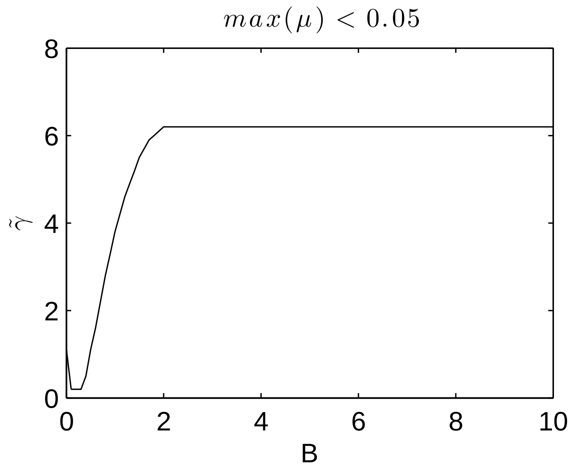

Figure 2. Minimal values of $\tilde{\gamma}$ for which $\mu < 0.05$ at any point of the stem as a function of B. The values of μ have been numerically computed for a wide range of values of B and $\tilde{\gamma}$ along the curvilinear abscissa s/L_{gz}. For each value of B, the minimal value of $\tilde{\gamma}$ for which μ is less than 0.05 everywhere along the organ is identified $max(\mu) < 0.05$. As long as $\tilde{\gamma} > 6.2$, the condition $\mu < 0.05$ is satisfied for any B at any point on the organ.

Movie 1. Solution of the $AC\dot{E}$ Model during exponential growth, $B = 10$, $\tilde{\gamma} = 0.1$. The color (from blue to red) codes for the absolute value of the curvature C(s,t). The simulated organ does not reach a steady state. The size of the curved zone are expanded by growth and the organ can not regulate its posture.

Movie 2. Solution of the $AC\dot{E}$ Model during exponential growth, $B = 10$, $\tilde{\gamma} = 10$. The color (from blue to red) codes for the absolute value of the curvature C(s,t). The simulated organ reaches a steady state even if the organ is elongating.

Movie 3. Solution of the $AC\dot{E}$ Model during subapical growth, $B = 10$, $\tilde{\gamma} = 0.1$. The color codes for the values of the median elongation rate $\dot{E}$ so that the organ is only elongating in the yellow part and not in the blue part (yellow $\dot{E} = \dot{E}_0$, blue $\dot{E} = 0$). As the simulated organ tries to reach the vertical, oscillations are fixed on the final shape due to the elements convected outside of the growth zone.

Movie 4. Solution of the $AC\dot{E}$ Model during subapical growth, $B = 10$, $\tilde{\gamma} = 10$. The color codes for the values of the median elongation rate $\dot{E}$ so that the organ is only elongating in the yellow part and not in the blue part (yellow $\dot{E} = \dot{E}_0$, blue $\dot{E} = 0$). The simulated organ reaches a steady state before the elements of the organ are convected outside of the growth zone. No oscillations are fixed on the final shape.
